# Liquid Chromatography-Tandem Mass Spectrometry Analysis Demonstrates a Decrease in Porins and Increase in CMY-2 β-Lactamases in *Escherichia coli* Exposed to Increasing Concentrations of Meropenem

**DOI:** 10.3389/fmicb.2022.793738

**Published:** 2022-02-28

**Authors:** Dimard E. Foudraine, Camiel N. M. Aarents, Agnes A. Wattel, Ria van Boxtel, Nikolaos Strepis, Marian T. ten Kate, Annelies Verbon, Theo M. Luider, Corné H. W. Klaassen, John Hays, Lennard J. M. Dekker, Jan Tommassen, Wil H. F. Goessens

**Affiliations:** ^1^Department of Medical Microbiology and Infectious Diseases, Erasmus University Medical Center (Erasmus MC), Rotterdam, Netherlands; ^2^Department of Molecular Microbiology, Institute of Biomembranes, Utrecht University, Utrecht, Netherlands; ^3^Department of Neurology, Neuro-Oncology Laboratory/Clinical and Cancer Proteomics, Erasmus University Medical Center (Erasmus MC), Rotterdam, Netherlands

**Keywords:** meropenem, *E. coli*, OmpC, OmpF, CMY-2-like, liquid chromatography-mass spectrometry, parallel reaction monitoring, antimicrobial resistance

## Abstract

While Extended-Spectrum β-Lactamases (ESBL) and AmpC β-lactamases barely degrade carbapenem antibiotics, they are able to bind carbapenems and prevent them from interacting with penicillin-binding proteins, thereby inhibiting their activity. Further, it has been shown that *Enterobacterales* can become resistant to carbapenems when high concentrations of ESBL and AmpC β-lactamases are present in the bacterial cell in combination with a decreased influx of antibiotics (due to a decrease in porins and outer-membrane permeability). In this study, a targeted liquid chromatography-tandem mass spectrometry (LC-MS/MS) assay was developed for the detection of the *Escherichia coli* porins OmpC and OmpF, its chromosomal AmpC β-lactamase, and the plasmid-mediated CMY-2 β-lactamase. *Bla*_CMY–2–like_ positive *E. coli* isolates were cultured in the presence of increasing concentrations of meropenem, and resistant mutants were analyzed using the developed LC-MS/MS assay, Western blotting, and whole genome sequencing. In five strains that became meropenem resistant, a decrease in OmpC and/or OmpF (caused by premature stop codons or gene interruptions) was the first event toward meropenem resistance. In four of these strains, an additional increase in MICs was caused by an increase in CMY-2 production, and in one strain this was most likely caused by an increase in CTX-M-15 production. The LC-MS/MS assay developed proved to be suitable for the (semi-)quantitative analysis of CMY-2-like β-lactamases and porins within 4 h. Targeted LC-MS/MS could have additional clinical value in the early detection of non-carbapenemase-producing carbapenem-resistant *E. coli*.

## Introduction

There are several antibiotic classes known, of which β-lactams are the most diverse and most frequently prescribed (Pandey and Cascella, 2020). β-lactam antibiotics can be categorized into several sub-classes, including the broad-spectrum carbapenem class. Carbapenems can withstand hydrolysis by most bacterial β-lactamases (including Extended-Spectrum β-Lactamases—ESBL) and are therefore considered one of the last resort antibiotics (Hawkey and Livermore, 2012). Unfortunately however, carbapenem-resistance rates are increasing worldwide (Miller and Humphries, 2016; Iovleva and Doi, 2017), with the increasing prevalence of carbapenem-resistant *Enterobacterales* (CRE) being especially worrying. Most CRE produce an acquired carbapenemase (Nordmann et al., 2011; Brolund et al., 2019). However, a second group of non-carbapenemase-producing CRE (non-CP-CRE) become resistant *via* a decrease in outer-membrane permeability combined with an increase in the production of other chromosomal or plasmid-encoded β-lactamases (Oteo et al., 2008; Adler et al., 2013; Goessens et al., 2013; Tangden et al., 2013; Miller and Humphries, 2016; Nordmann and Poirel, 2019). Some studies have even shown that up to 60% of carbapenem-resistant *Escherichia coli* in European hospitals comprised non-CP-CRE isolates (Grundmann et al., 2017; Nordmann and Poirel, 2019).

In *E. coli*, OmpC and OmpF are the two major porins responsible for the influx of β-lactams across the outer membrane (Jaffe et al., 1982; Choi and Lee, 2019). Alterations in the constriction regions of these porins can reduce permeation efficiency of β-lactams (Delcour, 2009; Vergalli et al., 2020). Further, a decrease in or complete loss of porin expression can also contribute to β-lactam resistance (Delcour, 2009; Vergalli et al., 2020). This decrease or absence of porins reduces the concentration of β-lactam molecules in the periplasm which can thereafter be bound and “trapped” by, for example, CMY-2-like β-lactamases (Goessens et al., 2013). Other β-lactamases that have been linked to carbapenem resistance in non-CP-CR *E. coli* include the class A β-lactamases TEM-1 and CTX-M, the class D β-lactamase OXA-1, as well as multiple class C β-lactamases including *E. coli* chromosomal AmpC (cAmpC) (Mammeri et al., 2008, 2010; Adler et al., 2013).

Unfortunately, no reliable phenotypic assays are currently available to specifically detect the absence of OmpC and OmpF. This information is important, as isolates which lack one or both of these main porins can rapidly become resistant to multiple antibiotics (including carbapenems) after antibiotic exposure (Pages et al., 2008; Goessens et al., 2013; van Boxtel et al., 2017; Vergalli et al., 2020). Additionally, the development of such an assay could aid in understanding the nature of carbapenem resistance in isolates that do not express carbapenemases. While real-time PCR assays are frequently used to confirm or screen for antimicrobial resistance mechanisms, they cannot detect a lack of porins, nor can they detect the actual production of β-lactamases. Although the relative abundance of proteins can be predicted based on mRNA using RT-qPCRs, porins are also regulated by RNA anti-sense regulators and by post-translational regulatory mechanisms which hamper quantitative prediction accuracy when using techniques based solely on detection of mRNA (Pages et al., 2008; Charretier et al., 2015b). In addition, frameshifts and premature stop codons are easily missed by RT-qPCR.

For the accurate detection and quantification of resistance mechanisms, and to predict their combined effect, a specific protein detection method would be more suitable. Before the molecular era, Western blotting was used to determine the presence of specific proteins, but nowadays MS-techniques are increasingly used for the detection of proteins. The use of matrix-assisted laser desorption/ionization time-of-flight (MALDI-TOF) mass spectrometry is widely implemented for bacterial identification in diagnostic medical microbiology laboratories, and its use has also previously been reported in research settings for the detection of AmpC-type β-lactamases (Hart et al., 2015; Espinosa et al., 2018), OmpC and OmpF porins (Hu et al., 2015), and several other resistance mechanisms (Oviano and Bou, 2019). However, the MALDI-TOF analyzer is primarily designed to generate a spectral fingerprint of the most abundant proteins and is less suited for the accurate identification, let alone quantification of less abundant protein markers (Intelicato-Young and Fox, 2013). In contrast, targeted liquid chromatography-tandem mass spectrometry (LS-MS/MS) offers increased sensitivity and specificity by separating and filtering peptides prior to detection. Therefore, targeted LC-MS/MS using a triple quadrupole or an Orbitrap mass spectrometer can be used for the accurate detection and quantification of many antimicrobial resistance mechanisms (Charretier et al., 2015a,b; Charretier and Schrenzel, 2016; Hassing et al., 2016; Wang et al., 2017, 2019; Cecchini et al., 2018; Foudraine et al., 2019, 2021a).

In the current study, we developed a single targeted LC-MS/MS assay for the detection and (relative) quantification of the *E. coli* porins OmpC and OmpF, as well as CMY-2-like and cAmpC β-lactamases. Subsequently, we cultured several clinical *E. coli* isolates carrying *bla*_CMY–2–like_ genes in the presence of meropenem to induce meropenem resistance *in vitro*. We then used the developed assay to analyze how the abundance of the porins and β-lactamases correlated to resistance, and in addition, we applied whole-genome sequencing (WGS) to place our findings within the context of the underlying genetic mechanisms.

## Materials and Methods

### Bacterial Isolates

Ethical approval was not required as only stored bacterial strains were used. A total of 15 clinical *E. coli* strains and two *E. coli* laboratory strains were used in this study. All strains were obtained from the Erasmus MC collection of bacterial isolates or had been used in the previously published study of van Boxtel et al. (2017). Twelve clinical strains were selected based on a phenotype which suggested the presence of plasmid-encoded AmpC-type β-lactamases with minimum inhibitory concentrations (MICs) of 2 mg/L or higher for third generation cephalosporins and MICs of 8 mg/L or higher for cefoxitin (Jacoby, 2009). The presence of CMY(-like) β-lactamase genes was confirmed by PCR using the primers described in the publication of Perez-Perez and Hanson (2002). In addition, three known cAmpC hyperproducers were included from a previous study (numbered 187, 188 and 190) (Foudraine et al., 2021b). Finally, the *E. coli* laboratory strain Top10F’ that expressed both OmpC and OmpF (Invitrogen, Thermo Fisher Scientific, Carlsbad, CA, United States) and the porin-deficient derivative CE1536 of strain BL21 (DE3) (Renault et al., 2012) were used as positive and negative control strains for the LC-MS/MS experiments. In addition, transformed derivatives of Top10F, i.e., Top10F’-p2761 and Top10F’-pmr42 which carried plasmids containing *bla*_CMY–2_ were used from a previous study as positive controls for Western blotting (van Boxtel et al., 2017).

### Selection of Meropenem Resistant Mutants

**FIGURE 1:**
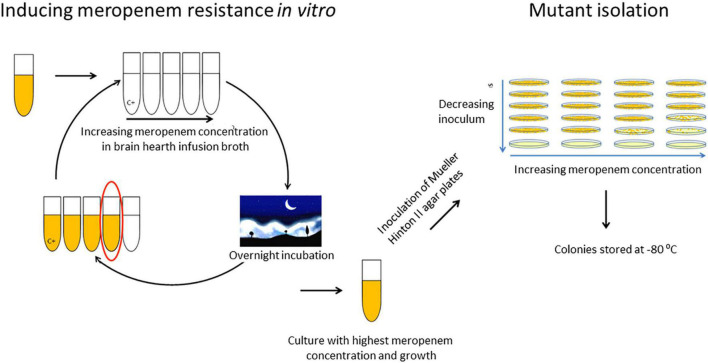
*In vitro* exposure to increasing meropenem concentrations and selection of mutants.

All *bla*_CMY–like_-positive isolates were initially susceptible to meropenem with MICs of 0.25 mg/L or lower (VITEK 2, BioMérieux). To study the effect of prolonged meropenem exposure on these strains, each strain was exposed to increasing concentrations of meropenem (Sigma Aldrich, St. Louis, MO, United States) over a period of multiple weeks (Figure 1). First, isolates were grown in 5 ml brain hearth infusion broth (BHI). Next, 2.5 ml was divided over four vials containing 4.5 ml BHI broth with increasing meropenem concentrations and one growth control vial without meropenem, and 2.5 ml was stored. Subsequently, the vials were incubated overnight and the broth of the vial containing the highest meropenem concentration in which bacterial growth was observed was again divided over four new vials with increasing meropenem concentrations. This procedure was subsequently repeated for 15–25 cycles until a maximum meropenem concentration was attained. Meropenem concentrations in the vials ranged from half of the previous concentration at which growth still occurred, and increased in steps of twofold to four times the previous concentration at which growth occurred. After every two to four cycles, and preferably after growth in a vial with a higher concentration of meropenem, the broth with the highest meropenem concentration was also serially diluted and inoculated on Mueller Hinton II (MHII) agar plates containing half to four times the meropenem concentration of the vial. After overnight incubation, single mutant colonies on the agar plate with the highest meropenem concentration were isolated and stored at −80°C. These experiments of prolonged meropenem exposure were performed in triplicate by three different technicians for all twelve clinical isolates. Meropenem MICs of all stored mutants were determined by broth microdilution.

### Liquid Chromatography-Tandem Mass Spectrometry Assay Development

Reference sequences of *bla*_cAmpC_ (from *E. coli* K-12), *bla*_CMY–2_, *ompC*, and *ompF* were obtained from the NCBI nucleotide RefSeq database^1^. To select tryptic peptides from conserved protein regions, variant sequences were obtained from the NCBI nucleotide collection^2^ which was accessed multiple times in September 2019. For cAmpC, OmpC, and OmpF, the search was restricted to *E. coli* sequences (Taxid: 562). All variant sequences were aligned and translated and sequences with less than 80% query coverage were removed. Tryptic peptides from 6 to 20 amino acids without a cysteine and preferably without a methionine or a double lysine/arginine motif at the N- or C-terminus (ragged end) were selected as candidate peptides (Supplementary Table 1). In addition to porin and β-lactamase peptides, internal quality control peptides were included from a previous study for verification of adequate sample pre-treatment (Foudraine et al., 2021a). General peptide uniqueness of all candidate peptides was assessed by performing BLASTp searches in the NCBI non-redundant protein sequence database. The coverage and specificity of the CMY-2-like and cAmpC candidate peptides were also assessed *in silico* by comparing all class C β-lactamases provided by the Beta-Lactamase Database (Naas et al., 2017). Subsequently, a targeted LC-MS/MS experiment was performed to select the best performing peptide markers. For this purpose, nine previously characterized *E. coli* isolates (Foudraine et al., 2021b) including three cAmpC hyperproducers, two CMY-2-like positive isolates, and the laboratory strain Top10F were used. The mass error, peak height, number of peptide fragments and peak shape were evaluated for all candidate peptides. For CMY-2-like and OmpF, two peptides were selected, for cAmpC three peptides, and for OmpC four peptides (Table 1). Stable isotope-labeled (SIL) peptides were ordered for all selected peptides (Pepscan, Lelystad, Netherlands). Single and fixed concentrations of SIL peptides were determined in a separate experiment using the original/parental clinical isolates and the *E. coli* laboratory strain Top10F’ to obtain a ratio between endogenous and SIL peptides close to one. Detection of all selected peptides was combined into a single multiplex assay. Peptides were considered detected when the ratio dot products (rdotp) was above 0.95 and if the mass error was < 10 ppm (Foudraine et al., 2019). Endogenous peptide to SIL peptide ratios were calculated using Skyline software (MacCoss Lab Software, University of Washington, United States) to compare the relative quantities of porin and CMY peptides between parental and mutant isolates.

**TABLE 1:** In total, 14 peptides were selected and included in a multiplex LC-MS/MS assay for the detection of the β-lactamases cAmpC and CMY-2-like, the porins OmpC and OmpF, and three “housekeeping” proteins for quality control purpose.

Protein	Peptide	Coverage of sequences from NCBI blast	Coverage of annotated sequences	β-lactamase variants missed / location in porin	Mass (m/z)	Retention window (min)	Fragment ions included
cAmpC	QPVTQQTLFE LGSVSK	1,170 / 1,179	376 / 405	14, 36, 75, 112, 113, 121, 169, 223, 237, 247, 248, 249, 281, 291, 297, 333, 374, 384–395	88,147,271	6.32–8.32	b3, y4, y5, y6, y7, y8, y9
	SSSDLLR	1,167 / 1,179	371 / 405	14, 20, 75, 112, 113, 121, 157–159, 169, 212, 225, 247–249, 254, 272, 281, 292, 325, 333, 374, 384–395	38,920,869	2.09–4.09	y3, y4, y5, y6
	NYPNPAR	1,170 / 1,173	364 / 405	14, 49, 56, 103, 104, 112, 113, 121–123, 169, 191, 209, 218, 219, 246–248, 258, 259, 278, 281, 306, 317, 329, 333, 355, 359, 374, 384–395	41,620,903	1.56–3.56	y3, y4, y5
CMY-2-like	TFNGVLGGDAIAR	925 / 950	150 / 154	36, 55, 60, 145	64,584,368	5.20–7.20	b4, y4,y6, y7, y8
	TGSTGGFGSYVA FVPEK	893 / 925	149 / 154	40, 45, 49, 144, 157	85,241,740	6.75–8.75	b3, y3, y4, y5, y6, y7, y8
OmpC	VAFAGLK	1,202 / 1,202		L3 constriction loop (AA 95–102)	35,321,833	3.67–5.67	b4, y3, y4, y5, y6
	VGSLGWANK	1,170 / 1,202		ß-sheet (AA 263–272)	46,625,343	3.46–5.46	b3, y3, y4, y5, y6, y7, y8
	FQDVGSFDYGR	1,148 / 1,202		L3 constriction loop (AA 103–113)	64,579,111	4.75–6.75	b3, y3, y4, y5, y6, y7, y8, y9
	GNGFATYR	1,117 / 1,202		ß-sheet (AA 145–153)	44,321,431	2.54–4.54	y3, y4, y5, y6
OmpF	AVGLHYFSK	1,122 / 1,125		ß-sheet (aa 39–47)	51,127,691	3.55–5.55	b3, y3, y4, y5, y7
	VGGVATYR	1,127 / 1,127		ß-sheet (aa 155–162)	41,172,705	1.90–3.90	b3, y3, y4, y5, y6, y7
Chaperone protein DnaK	SLGQFNLDGI NPAPR				79,991,790	6.61–8.61	b4, y4, y5, y6, y7 y8
30S ribosomal protein	GATVELADGV EGYLR				77,539,647	6.91–8.91	b3, y4, y5, y6, y7, y8
RNA polymerase	VADLFEAR				46,074,544	4.37–6.37	b3, y4, y5, y6, y7

*All peptides were measured with a charge of 2+. Coverage of each peptide was assessed by comparing variant sequences of cAmpC, CMY-2-like, OmpC and OmpF. Variant sequences were obtained by BLASTn searches using the reference sequences and the NCBI nucleotide collection (nr/nt) database. In addition, coverage of the peptides selected for cAmpC and CMY-2-like was also assessed by comparing the annotated sequences from the Beta-Lactamase Database (Naas et al., 2017).*

### Culturing, Lysis and Digestion Prior to Liquid Chromatography-Tandem Mass Spectrometry

Original *E. coli* isolates and mutants stored at −80°C were thawed and subcultured on Trypticase™ Soy Agar II plates supplemented with 5% sheep blood (Becton Dickinson, New Jersey, United States) and used to inoculate 4 ml of MH broth. After culturing for at least 5 h at 37°C and 150 rpm, suspensions were diluted using MH broth to an absorbance of 0.98–1.02 at 600 nm wavelength (OD600 DiluPhotometer™, München, Germany). Thereafter, 1 ml was transferred and centrifuged for 5 min at 21,000 *g*. Pellets were washed once with 500 μl of phosphate-buffered saline solution (pH 7.3 ± 0.2, NaCl 8.0 g/L, KCl 0.2 g/L, Na_2_HPO_3_ 1.15 g/L, KH_2_PO_4_ 0.2 g/L) and stored at −20°C. After thawing, bacterial pellets were suspended in 100 μl of water with 5% sodium deoxycholate and 7.5 mM dithiothreitol and SIL peptides were added (final concentrations in sample digest of 5 fmol/μl for CMY-2-like, OmpF and OmpC peptides, and 2.5 fmol/μl for cAmpC peptides). Samples were then sonicated for 5 min (Branson 2510 Ultrasonic Cleaner, Branson Ultrasonics, Danbury, United States) and incubated for 10 min at 80°C, 450 rpm. Next, 900 μl of water, 50 μl of Tris–HCl buffer (50 mM, pH 8) and 10 μl of trypsin (1 μg/μl) (Worthington, NJ, United States) were added, and samples were digested for 2 h at 37°C and 450 rpm. Subsequently, 30 μl of 5% trifluoroacetic acid was added, and samples were incubated for 5 min at 37°C and 450 rpm. Finally, samples were centrifuged for 10 min at 21,000 *g* and 4°C, and 500 μl of the supernatant was stored at −20°C.

### Liquid Chromatography-Tandem Mass Spectrometry

Stored digests were thawed and loaded onto Evotips (Evosep, Odense, Denmark) according to the manufacturer’s instructions and as previously described (Bache et al., 2018; Foudraine et al., 2019). LC-MS/MS was performed using the Evosep One (Evosep, Odense, Denmark) coupled to an Orbitrap mass spectrometer (Q Exactive HF Hybrid Quadrupole-Orbitrap, Thermo Fisher Scientific, Bremen, Germany). LC was performed using the manufacturer’s separation method of 11.5 min (100 samples/day) (Bache et al., 2018). The Q Exactive HF system was operated in parallel reaction monitoring (PRM) mode. The following settings were used: a quadrupole isolation window of 0.6 m/z units, an automatic gain control target value of 1 × 10^6^ ions, a maximum fill time of 150 ms and a resolving power of 30,000 at 400 m/z. A normalized collision energy of 27% was used for all peptides. Measurements were unscheduled in the peptide selection experiments. Measurements were scheduled with retention windows of 2 min for each peptide in the subsequent experiments. LC-MS/MS data was analyzed in Skyline daily 19.1 or later (MacCoss Lab Software, University of Washington, United States). The mass spectrometry proteomics data have been deposited to the n ProteomeXchange Consortium via the PRIDE (Perez-Riverol et al., 2019) partner repository with the dataset identifier PXD025363.

### Western Blotting

For the preparation of whole cell lysates, bacteria were cultured at 37°C in lysogeny broth. For the detection of porins, cells were collected by centrifugation from overnight grown cultures, resuspended in 50 mM Tris–HCl, 5 mM EDTA, pH 8.5, and disrupted by ultrasonication (Branson B-12, Emerson, St. Louis, MO, United States). For the detection of β-lactamases, cells from exponentially growing cultures were first converted to spheroplasts and then disrupted by sonication after a freeze-thaw cycle as described (Paltansing et al., 2015). Sodium dodecyl sulfate-polyacrylamide gel electrophoresis (SDS-PAGE) analysis of the lysates was performed according to Laemmli (1970). To improve the separation of the porins OmpC and OmpF, gels were used containing 5 M urea. For Western blot analysis of the porins OmpC and OmpF, a polyclonal antiserum raised against the *E. coli* porin PhoE was used that was previously shown to cross-react with OmpC and OmpF (Goessens et al., 2013). The *E. coli* cAmpC and CMY-2-like β-lactamases were detected using antisera previously described (Goessens et al., 2013; van Boxtel et al., 2017).

### Whole Genome Sequencing

Genomes of the original isolates and selected mutants were sequenced by Novogene (Beijing, China) using an Illumina HiSeq X Ten system. The genomes were assembled using Unicycler v0.4 with default parameters (Wick et al., 2017). A cgMLST analysis was performed for *E. coli* strains based on SeqSphere+^3^ to assess isolate heterogeneity and rule out contamination. To screen for the presence of additional β-lactamase genes, all genome sequences were analyzed using ABRicate^4^ and the CARD database, version 3.0.9 (Alcock et al., 2020). The coverage of contigs containing *bla*_CMY–2–like_ and *bla*_CTX–M–15_ genes were compared to the coverage of chromosomal contigs in each isolate using the coverage depths calculated by the Unicycler and CLC (Qiagen, Hilden, Germany) assemblers.

### Mutation Analysis

Coding sequences for the investigated genes were extracted from the genomic assemblies using the sequence extraction tool in BioNumerics v7.6 software (Applied Maths, St.-Martens-Latem, Belgium). Reference sequences for the extraction were taken from the genome of *E. coli* MG1655 (NC_000913). Extracted sequences were translated and the protein sequences were compared to that of the parent strain to identify amino-acid changes. If no full-length coding sequence could be extracted, the structural integrity of the gene was verified by read-mapping using the CLC Genomics Workbench (Qiagen, Hilden, Germany). Mapped reads were visually inspected for issues (Supplementary Figure 1).

## Results

### General Peptide Uniqueness

Peptides were selected for the detection of CMY-2-like, cAmpC, OmpC and OmpF using LC-MS/MS (Table 1). To assess if the peptides were specific, *in silico* specificity analysis was performed for all candidate peptides, and the results are described here for the selected peptides. The peptide SSSDLLR (cAmpC) was also present in a mechanosensitive ion channel of *Burkholderia oklahomensis*. The peptide NYPNPAR (cAmpC) was also present in the class C β-lactamase EDC-1 of *Edwarsiella tarda* (Naas et al., 2017). The peptide TEQQIADIVNR (CMY-2-like) was also present in the CMY-2-like similar class C β-lactamase EC-68 of an uncultured bacterium (Naas et al., 2017). The selected OmpC and OmpF peptides were not present in non-porin proteins in *Enterobacterales*. However, there is significant homology between porin sequences from different *Enterobacterales* spp., and the selected OmpC and OmpF peptides were also present in OmpC and OmpF orthologs of other bacterial species.

### Selection of Meropenem-Resistant Mutants

Of the 12 strains cultured under increasing meropenem concentrations, four strains showed an approximate 100-fold increase in meropenem MICs resulting in MICs of 4 mg/L or higher. This trend was found in all three replicate experiments. Three strains showed a similar increase, but in only one out of the three replicate experiments, and no increase or a moderate 5 to 10-fold increase in the two other replicate experiments. Experiments for the remaining strains showed a moderate increase in MICs or no increase at all. For the subsequent analysis, the strains that became resistant to meropenem (MICs ≥ 8 mg/L) were selected in order to analyze the underlying mechanisms. Therefore, one replicate series was selected for each of the four strains that showed significant MIC increases in all replicate experiments (R1281, B2591, R2723, R2761), one replicate series of strain B2582 was selected that showed a significant increase in MICs while the other replicate series did not, and one replicate series was selected which only showed a modest increase in MICs (R1810). Key characteristics of the selected strains and their mutants are shown in Table 2. None of the strain carried carbapenemase genes as determined by WGS.

**TABLE 2:** Characteristics of all mutants and identified causes for the loss of OmpC and/or OmpF, and the increase in CMY-2-like β-lactamases at the DNA level.

Strain	β-lactamase genes present	Mutant number	MEM MIC	Mutations in OmpC	Mutations in OmpF	Coverage of *bla*_CMY–2–like_	Coverage of *bla*_CTX–M–15_
R2723	*bla*_CMY–2_ *bla*_TEM–1_	Original isolate	≤0.063	-	-	1.7x	N.A.
		Mutant #1	0.5	Q267X	-	1.8x	N.A.
		Mutant #2	1	XXX-1	-	2.0x	N.A.
		Mutant #3	4	Q376X	-	11.1x	N.A.
		Mutant #4	32	XXX-2	XXX-3	11.1x	N.A.
		Mutant #5	32	XXX-2	XXX-3	12.5x	N.A.
		Mutant #6	32	XXX-2	XXX-3	11.4x	N.A.
		Mutant #7	>32	XXX-2	XXX-3	12.4x	N.A.

R2761	*bla*_CMY–42_ *bla*_CTX–M–15_ *bla*_OXA–1_	Original isolate	≤0.063	-	-	0.8x	2.1x
		Mutant #1	2	Y152X	-	0.8x	2.2x
		Mutant #2	8	Y152X	-	0.8x	6.9x
		Mutant #3	16	Y152X	-	0.6x	5.2x
		Mutant #4	32	Y152X	-	0.7x	10.3x
		Mutant #5	32	Y152X	-	0.7x	10.8x
		Mutant #6	32	Y152X	-	0.6x	9.5x

B2591	*bla*_CMY–2_	Original isolate	≤0.063	-	-	0.7x	N.A.
		Mutant #1	≤0.063	-	-	1.2x	N.A.
		Mutant #2	≤0.063	-	-	1.1x	N.A.
		Mutant #3	0.25	Y344X	-	0.7x	N.A.
		Mutant #4	8	Y344X	XXX-4	6.0x	N.A.
		Mutant #5	16	Y344X	XXX-5	9.4x	N.A.
		Mutant #6	32	Y344X	XXX-5	4.8x	N.A.
		Mutant #7	32	Y344X	XXX-5	9.9x	N.A.

R1281	*bla*_CMY–2_ *bla*_TEM–1_	Original isolate	≤0.063	-	-	0.7x	N.A.
		Mutant #1	≤0.063	-	-	0.8x	N.A.
		Mutant #2	≤0.063	-	-	0.7x	N.A.
		Mutant #3	≤0.063	-	-	0.7x	N.A.
		Mutant #4	≤0.063	-	-	0.8x	N.A.
		Mutant #5	1	Q104X	-	0.8x	N.A.
		Mutant #6	2	Q104X	-	1.6x	N.A.
		Mutant #7	8	Q104X	-	8.0x	N.A.

B2582	*bla*_CMY–2_ *bla*_TEM–1_	Original isolate	≤0.063	-	-	1.6x	N.A.
		Mutant #1	0.125	-	-	3.0x	N.A.
		Mutant #2	0.125	-	-	2.8x	N.A.
		Mutant #3	1	Q171X	-	3.1x	N.A.
		Mutant #4	4	Q171X	-	14.6x	N.A.
		Mutant #5	4	Q171X	-	15.2x	N.A.
		Mutant #6	32	-	-	18.0x	N.A.

R1810 (control)	*bla*_CMY–2_ *bla*_TEM–1_	Original isolate	≤0.063	-	-	1.8x	N.A.
		Mutant #1	≤0.063	-	-	2.0x	N.A.
		Mutant #2	0.125	-	-	2.6x	N.A.
		Mutant #3	0.125	-	-	3.3x	N.A.
		Mutant #4	0.125	-	-	3.6x	N.A.
		Mutant #5	0.125	-	-	4.3x	N.A.
		Mutant #6	0.125	-	-	2.4x	N.A.
		Mutant #7	0.5	-	-	2.2x	N.A.

*MICs, minimum inhibitory concentrations shown in mg/L; MEM, Meropenem. Mutations resulting in a premature stop codon are indicated by the original amino acid followed by the location and an X. Gene interruptions are indicated with XXX and a random number to identify interruptions at different positions within the gene. The coverage of contigs containing bla_CMY–2–like_ and bla_CTX–M–15_ genes were compared to the coverage of chromosomal contigs in each isolate.*

### Protein Analysis of Mutant Isolates

**FIGURE 2:**
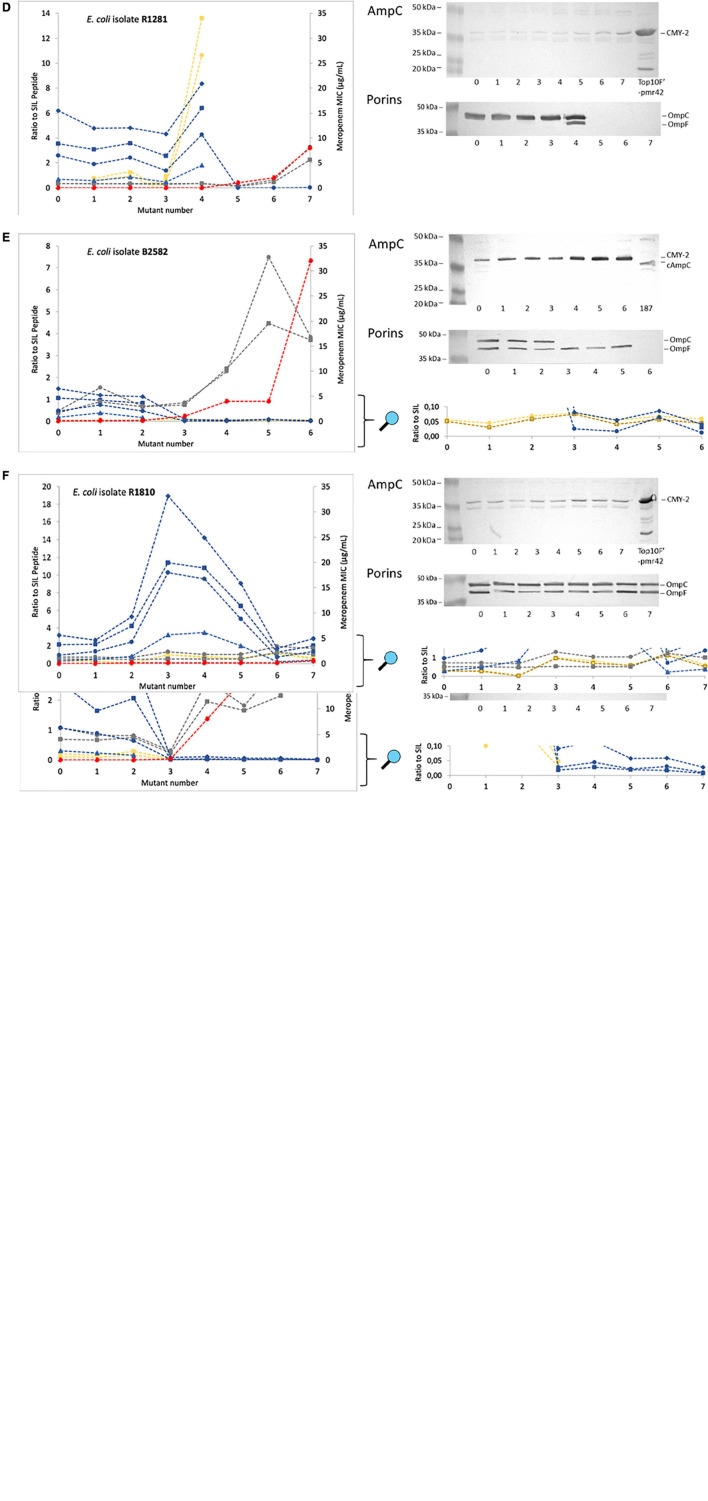
Analysis of the presence of CMY-2-like, cAmpC, and the porins OmpC and OmpF in mutant isolates of different *E. coli* strains selected under increasing meropenem concentrations using LC-MS/MS and Western blotting. On the Y-axis the meropenem minimum inhibitory concentration and mass spectrometry signal intensity of each endogenous peptide divided by signal intensity of each stable isotope labeled (SIL) peptide. When a peptide was not detected in a mutant isolate, the corresponding symbol was left out. All detected peptides were shown even when ratios to SIL approached zero. Molecular weight standard proteins are indicated (in kDa) at the left of the blots. Strains Top10F’-p2761 and Top10F’-pmr42 were used in the Western blot analysis as positive controls for CMY-2 and strain 187 as a positive control for cAmpC. **(A–F)** Represent the different clinical strains referred to throughout the “Results” section.

A significant decrease of OmpC was observed in all five selected series that developed meropenem resistance (Figures 2A–E), but not in the control series (Figure 2F). The first mutants of series R2723 and R2761 already showed a decrease in OmpC, observed by both LC-MS/MS and Western blot analysis (Figures 2A,B). The other three series, i.e., B2591, R1281 and B2582 showed a delayed decrease of OmpC (Figures 2C,D,E). In all mutant isolates in which OmpC was detected on Western blot, all four selected peptides were detected by LC-MS/MS as well. In addition, in the mutant isolates in which OmpC was not detected on Western blot, one to three peptides of OmpC were still detected at low ratios and only when mutations resulting in premature stop codons occurred after the corresponding peptide coding regions (Tables 1, 2). The OmpC peptide VAFAGLK was even detected in all mutant isolates. OmpC remained clearly detectable in the control series R1810 by both Western blot analysis and LC-MS/MS analysis. Remarkably, OmpC abundance even increased in mutants 2–5 of this series suggesting an increase in expression (Figure 2F).

OmpF was only detected by LC-MS/MS in the first three mutants of series R2723 and B2591 but not on Western blot. In strain R2761, only one peptide of OmpF was detected in mutants 1 and 2. Interestingly, OmpF expression increased in mutant 4 of series R1281 (Figure 2D) as observed by both LC-MS/MS and Western blotting, while both porins were less abundant or absent in subsequent mutants. In series B2582, OmpF was detected in all mutants using LC-MS/MS with low endogenous to SIL peptide ratios of around 0.05, whilst it was abundantly detected by Western blot analysis, except in mutant six (Figure 2E). In the control series, OmpF was detected in all mutants by both LC-MS/MS and Western blot analysis.

After a decrease in OmpC and OmpF, CMY-2 β-lactamase abundance increased with a concomitant increase in meropenem MICs in the series R2723, B2591, R1281 and B2582. A similar increase in meropenem MICs was observed in series R2761 while CMY-2 abundance did not increase (Figure 2B). In the control series R1810, neither the CMY-2 abundance nor the meropenem MICs increased. Interestingly, in contrast to CMY-2-like, cAmpC was not detected by LC-MS/MS in any of the original isolates nor in the mutants derived from them, but it was detected in the cAmpC positive control strain (data not shown). This indicated that the increase in meropenem MICs of the mutants was not caused by an increase in cAmpC production.

### Genetic Analysis of Mutant Isolates

To analyze the underlying genetic alterations possibly responsible for the decrease in porins and increase in CMY-2 β-lactamases, WGS analysis was performed on the parental and mutant isolates (Table 2). Mutations in *ompC* genes resulting in premature stop codons or *ompC* gene interruptions coincided with modest increases in meropenem MICs in all strains, except in the control strain R1810 in which no mutations in *ompC* occurred. In strain R2723, different mutations were responsible for the loss of OmpC in different mutants. A mutation resulting in a stop codon (Q267X) was observed in the first mutant, a gene interruption was observed in the second mutant, and a mutation resulting in Q376X which only resulted in the loss of the last three OmpC amino acids was observed in the third mutant. A different gene interruption was observed in mutants four to seven. In strains R2761, B2591, and R1281, the mutations resulting in Y152X, Y344X, and Q104X were responsible for the loss of OmpC, respectively. Finally, in strain B2582, a mutation resulting in Q171X was responsible for the loss of OmpC, but remarkably this mutation was not observed in the last mutant. In addition, no other mutations that could explain the loss of OmpC were found in the *ompC* gene of this mutant.

Of the three strains that lost OmpF, gene interruptions were responsible for its loss in strain R2723 and B2591. In strain B2591, one gene interruption occurred in mutant four, while a different interruption was observed in mutants five to seven. In strain R1281, no mutations were detected in the *ompF* gene that accounted for the sudden increase and subsequent loss of OmpF.

In addition to porin genes, *bla*_CMY–2–like_ genes were analyzed in the original isolates and in their mutants to reveal the mechanisms responsible for the observed increases in CMY-2-like β-lactamases (Table 2). All *bla*_CMY–2–like_ genes appeared to be located on plasmids or were flanked by an insertion sequence element and BLAST analysis of the corresponding contigs showed near complete similarity to known plasmid sequences. No mutations were demonstrated in the promoter regions of the *bla*_CMY–2–like_ genes indicating that an increase in gene copy numbers, e.g., by loss of plasmid copy-number control (van Boxtel et al., 2017), was most likely responsible for the increase in CMY-2 β-lactamases. This was confirmed by comparing the *bla*_CMY–2–like_ gene coverage in each mutant with the coverage in the original isolate. All final mutants that showed an in increase in CMY-2 abundance also showed an increase in gene coverage by at least sevenfold. In strain R2761, no increase in coverage of *bla*_CMY–2–like_ occurred; however, a 4.5-fold increase in coverage of *bla*_CTX–M–15_ was observed in the final mutant of strain R2761 compared to the coverage in the original isolate.

## Discussion

The increasing prevalence of CRE is threatening the success of β-lactam antibiotics (Brolund et al., 2019). While carbapenemases are the usual cause of carbapenem resistance, a decrease in membrane permeability and a concomitant increase of certain non-carbapenemase β-lactamases can also cause carbapenem resistance in *Enterobacterales* (Oteo et al., 2008; Adler et al., 2013; Goessens et al., 2013; Tangden et al., 2013; Miller and Humphries, 2016; Nordmann and Poirel, 2019). Several authors have previously described carbapenem resistance in *E. coli* with ESBL and/or AmpC-type β-lactamase genes that lack porins OmpC and/or OmpF (Oteo et al., 2008; Yang et al., 2010; Wozniak et al., 2012; Tangden et al., 2013; Ho et al., 2016; Nuramrum et al., 2017; Chang et al., 2019; Tian et al., 2020). Others have further characterized isolates in which this occurred and performed additional genetic experiments (Mammeri et al., 2008; Girlich et al., 2009; Adler et al., 2013; Goessens et al., 2013; van Boxtel et al., 2017). However, non-CP-CRE have never been studied using an advanced protein detection method such as LC-MS/MS. Furthermore, while several techniques are available for both the detection of carbapenemase activity (Tamma and Simner, 2018) as well as for the identification of specific carbapenemases (Boutal et al., 2018; Traczewski et al., 2018), no accepted assay is currently available to detect (a lack of) porins in routine diagnostic medical microbiology laboratories. This is unfortunate, as a lack of porins together with the presence of non carbapenemase β-lactamases is responsible for carbapenem resistance in a significant number of *E. coli* and other *Enterobacterales* (Nordmann and Poirel, 2019). Rapid detection of this combination could aid in earlier selection of optimal antibiotic therapy that is not affected by a lack of porins and/or the presence of AmpC-type β-lactamases.

In the current study, several clinical *E. coli* isolates were cultured under increasing meropenem concentrations, and the underlying mechanisms of the resulting resistant mutants were studied using a newly developed LC-MS/MS assay, Western blotting, and WGS. First, we used a genoproteomic approach to select tryptic peptides for the detection of the *E. coli* porins OmpC and OmpF, and for the β-lactamases CMY-2-like and cAmpC. Since genes are prone to mutations, conserved protein regions were selected in which these mutations do not frequently occur. Further, specificity was assessed for all porin peptides to assure correct identification of the porins of interest. For CMY-2-like, two peptides were selected that together covered all CMY-2-like β-lactamases. Both peptides were previously used for the detection of CMY-2-like as well, albeit using MALDI-TOF mass spectrometry (Hart et al., 2015). For cAmpC, the three selected peptides together covered 373 of the 395 annotated AmpC variants in the Beta-Lactamase Database (Naas et al., 2017). Detection of all of the selected peptides and additional SIL peptides was combined into a single multiplex assay with a time-to-result of less than 4 h.

When peptides for other resistance mechanisms, such as ESBLs, carbapenemases (Foudraine et al., 2019), fluoroquinolone resistance mechanisms (Hassing et al., 2016), and aminoglycoside resistance mechanisms (Foudraine et al., 2021a) would be added to the current panel of CMY, *E. coli* cAmpC, OmpC and OmpF, a single multiplex LC-MS/MS assay would be able to detect several prevalent mechanisms that confer resistance to beta-lactams, fluoroquinolones and aminoglycosides in for instance *E. coli*. While such an assay could be used to confirm or rule out the presence of specific resistance mechanisms after routine susceptibility testing, it may be more beneficial to use it as a rapid screening test for important liquid cultures such as blood cultures. If the currently used protocol would be automated, such an assay could compete in turnaround time with other assays used for blood cultures including rapid phenotypic and genotypic methods (Idelevich and Becker, 2019; Banerjee and Humphries, 2021). In addition, it would offer the ability to detect proteins semi-quantitatively which offers unique information compared to other techniques (Charretier and Schrenzel, 2016; Wan Nur Ismah et al., 2018; Foudraine et al., 2021b).

To study the sequential development of carbapenem resistance in *E. coli* carrying *bla*_CMY–2–like_ genes and to evaluate the ability of the developed LC-MS/MS assay to simultaneously detect CMY-2 and porins, we exposed twelve *bla*_CMY–2–like_ positive strains to increasing meropenem concentrations and selected six strains for further analysis. A comparable selection procedure has previously been demonstrated to result in mutations similar to those found in clinical non-carbapenemase producing meropenem-resistant *E. coli* (van Boxtel et al., 2017). In all of our *E. coli* strains that developed meropenem resistance, a decrease in porin abundance was observed by LC-MS/MS which coincided with interruptions or premature stop codons in the corresponding genes. The timing of this occurrence differed as for instance strains R2723 and R2761 lost their porin OmpC significantly earlier during the selection procedure than strains B2582 and B2591. These differences might be explained by the random timing of mutation events. Alternatively, it could also be hypothesized that bacteria need a certain genomic background in order to overcome a drastic event, such as the sudden loss of porins, which is expected to lead to nutritional deficiency (Ferenci, 2005). If bacteria are not prepared for such an event through mutational adaptation, they will experience a severe growth defect, and they will quickly be outcompeted by fitter strains (Adler et al., 2013; Knopp and Andersson, 2015). Another explanation may be the number of porins initially present. Strains with a single porin are more likely to lose this porin when this is advantageous than strains with two or more porins as the effect of losing one of two porins will not offer much benefit in inhibiting β-lactam uptake. Furthermore, the loss of two or more porins caused by mutational events at the same time is not that likely.

Gene interruptions in the mutants studied were most likely caused by insertion sequence elements in the coding regions of *ompC* and/or *ompF* (Fernandez and Hancock, 2012; van Boxtel et al., 2017). Unfortunately, this could not be confirmed in the current analysis due to the use of short-read sequencing.

In four of the five strains that became meropenem resistant, the increase in CMY-2 β-lactamase coincided with an increase in gene copy numbers as demonstrated by an increase in gene-coverage. Interestingly, the decrease in OmpC preceded the increase of CMY-2 in all four strains. The necessity of a decrease in OmpC prior to an increase in CMY-2-like was shown by the control series R1810. In this series, no decrease in OmpC was observed and it could be hypothesized that some tolerability to meropenem is required before additional mutations can occur. In mutant series R2761, a decrease in OmpC preceded an increase in meropenem MICs but an increase in CMY-2-like abundance was not observed by LC-MS/MS. This was in contrast to the results of a previous study, which did show an increase in CMY-2 abundance due to the loss of plasmid copy-number control when meropenem resistance developed in strain R2761 (van Boxtel et al., 2017). Differences between the previous and the current study could be explained by the different mutant selection procedures used. In the current study, single colonies were used that derived from an ongoing liquid culture which resulted in different subpopulations with distinct mutations. Furthermore, the stored colonies were thawed and sampled multiple times to obtain fresh cultures for LC-MS/MS, WGS and Western blot analysis which could have resulted in additional discrepancies. In contrast to the other isolates, strain R2761 also carried a *bla*_OXA–1_ and a *bla*_CTX–M_ gene, which together with the decrease in porins could have played a role in development of meropenem resistance. An increase in CTX-M and a decrease in porins was also previously demonstrated to result in meropenem resistance in *E. coli* (Adler et al., 2013). Expression of chromosomal *ampC* was neither observed by LC-MS/MS nor by Western blotting in any of the mutants. This could be due to the fact that these strains already expressed CMY-2-like β-lactamases, and there are several DNA alterations required before regular *E. coli* turn into cAmpC hyperproducers (Livermore, 1995; Paltansing et al., 2015), and perhaps exposure to meropenem does not select for chromosomal AmpC mutants.

Overall, most LC-MS/MS observations were in agreement with the results of the Western blot analysis, however, some observed differences are worth mentioning. The peptide VAFAGLK (OmpC) was detected in all isolates, even when the porin was no longer detected on Western blot. This indicated that the part of *ompC* before a premature stopcodon or gene interruption was still transcribed and translated, and that some peptides from this part could still be detected using the highly sensitive LC-MS/MS assay. Only when all four OmpC peptides were detected, the porin was also detected by Western blotting. In general, OmpF abundance was significantly lower than OmpC abundance, and in some of the mutants, OmpF was only detected by LC-MS/MS. In strain B2582, Western blot analysis most likely detected another porin instead of OmpF (e.g., NmpC, OmpN, PhoE or a porin encoded by a prophage) (Nikaido, 2003) as it was no longer detected in mutant six (as opposed to LC-MS/MS results), and LC-MS/MS ratios for OmpF peptides were only around 0.05 while Western blot analysis showed clear bands. Apparently, this porin was important for the uptake of meropenem, as meropenem MICs increased substantially after its loss.

The current study is in line with previous studies which demonstrated that a decrease in porins and an increase in AmpC-type β-lactamases results in carbapenem resistance in *E. coli* (Mammeri et al., 2008, 2010; Oteo et al., 2008; Goessens et al., 2013; van Boxtel et al., 2017). In addition, this study shows that LC-MS/MS can be applied for the rapid semi-quantitative analysis of CMY-2-like β-lactamases and porins, which could be useful in the routine diagnostic medical microbiology laboratory for the early detection of non-CP-CRE and the differentiation from CP-CRE.

## Data Availability Statement

The datasets presented in this study can be found in online repositories. The names of the repository/repositories and accession number(s) can be found below: https://www.ebi.ac.uk/ena, PRJEB44241, ERP128274; http://www.proteomexchange.org/, PXD025363.

## Author Contributions

WG, JT, DF, AW, CK, and TL designed the study. CA, DF, AW, RB, MK, and LD performed and/or supervised the laboratory work. CA, DF, AW, RB, CK, NS, LD, JT, and WG analyzed the data. NS and LD curated the data in online repositories. WG, JT, TL, CK, JH, and AV supervised the study. WG and TL obtained project funding. WG performed the project administration. CA, AW, RB, CK, and DF visualized the data. DF and CA wrote the first version of the manuscript. All authors edited and approved the manuscript.

## Conflict of Interest

The authors declare that the research was conducted in the absence of any commercial or financial relationships that could be construed as a potential conflict of interest.

## Publisher’s Note

All claims expressed in this article are solely those of the authors and do not necessarily represent those of their affiliated organizations, or those of the publisher, the editors and the reviewers. Any product that may be evaluated in this article, or claim that may be made by its manufacturer, is not guaranteed or endorsed by the publisher.

## Footnotes

1
https://www.ncbi.nlm.nih.gov/nucleotide/

2
https://blast.ncbi.nlm.nih.gov/Blast.cgi

3
https://www.ridom.de/seqsphere/

4
https://github.com/tseemann/abricate

## Supplementary Material

Supplementary Figure 1 Application of read-mapping to check the structural integrity of genes. The example shows the read-mapping results of the B2591 parent strain and its mutants for the *ompF* gene using the gene from *E. coli* MG1655 plus 100 nucleotide flanking sequences as a reference. Colors in the cumulative coverage graph and from individual reads are as follows. Dark-blue: forward paired read; light blue: reverse paired read; green: forward unpaired read; red: reverse unpaired read. The vertical colored lines indicate sequence variations relative to the reference sequence. Below the cumulative coverage graph, a fraction of the individual reads are shown. The red oval indicates regions where the structural integrity of the gene was affected. In mutant 4, the gene was affected at a different position than in mutants 5–7.

## References

[B1] Adler M. Anjum M. Andersson D. I. Sandegren L. (2013). Influence of acquired beta-lactamases on the evolution of spontaneous carbapenem resistance in *Escherichia coli*. *J. Antimicrob. Chemother.* 68 51–59. 10.1093/jac/dks368 22977158

[B2] Alcock B. P. Raphenya A. R. Lau T. T. Y. Tsang K. K. Bouchard M. Edalatmand A. (2020). CARD 2020: antibiotic resistome surveillance with the comprehensive antibiotic resistance database. *Nucleic Acids Res.* 48 D517–D525. 10.1093/nar/gkz935 31665441 PMC7145624

[B3] Bache N. Geyer P. E. Bekker-Jensen D. B. Hoerning O. Falkenby L. Treit P. V. (2018). A Novel LC system embeds analytes in pre-formed gradients for rapid, ultra-robust proteomics. *Mol. Cell Proteomics* 17 2284–2296. 10.1074/mcp.TIR118.000853 30104208 PMC6210218

[B4] Banerjee R. Humphries R. (2021). Rapid antimicrobial susceptibility testing methods for blood cultures and their clinical impact. *Front. Med. (Lausanne)* 8:635831. 10.3389/fmed.2021.635831 33777978 PMC7987685

[B5] Boutal H. Vogel A. Bernabeu S. Devilliers K. Creton E. Cotellon G. (2018). A multiplex lateral flow immunoassay for the rapid identification of NDM-, KPC-, IMP- and VIM-type and OXA-48-like carbapenemase-producing *Enterobacteriaceae*. *J. Antimicrob. Chemother.* 73 909–915. 10.1093/jac/dkx521 29365094 PMC5890661

[B6] Brolund A. Lagerqvist N. Byfors S. Struelens M. J. Monnet D. L. Albiger B. (2019). Worsening epidemiological situation of carbapenemase-producing *Enterobacteriaceae* in Europe, assessment by national experts from 37 countries, July 2018. *Euro Surveill.* 24:1900123. 10.2807/1560-7917.ES.2019.24.9.1900123 30862330 PMC6402177

[B7] Cecchini T. Yoon E. J. Charretier Y. Bardet C. Beaulieu C. Lacoux X. (2018). Deciphering multifactorial resistance phenotypes in *Acinetobacter baumannii* by genomics and targeted label-free proteomics. *Mol. Cell Proteomics* 17 442–456. 10.1074/mcp.RA117.000107 29259044 PMC5836370

[B8] Chang Y. T. Siu L. K. Wang J. T. Wu T. L. Chen Y. H. Chuang Y. C. (2019). Resistance mechanisms and molecular epidemiology of carbapenem-nonsusceptible *Escherichia coli* in Taiwan, 2012-2015. *Infect. Drug Resist.* 12 2113–2123. 31406467 10.2147/IDR.S208231 PMC6642643

[B9] Charretier Y. Schrenzel J. (2016). Mass spectrometry methods for predicting antibiotic resistance. *Proteomics Clin. Appl.* 10 964–981. 10.1002/prca.201600041 27312049

[B10] Charretier Y. Kohler T. Cecchini T. Bardet C. Cherkaoui A. Llanes C. (2015b). Label-free SRM-based relative quantification of antibiotic resistance mechanisms in *Pseudomonas aeruginosa* clinical isolates. *Front. Microbiol.* 6:81. 10.3389/fmicb.2015.00081 25713571 PMC4322712

[B11] Charretier Y. Dauwalder O. Franceschi C. Degout-Charmette E. Zambardi G. Cecchini T. (2015a). Rapid bacterial identification, resistance, virulence and type profiling using selected reaction monitoring mass spectrometry. *Sci. Rep.* 5:13944. 10.1038/srep13944 26350205 PMC4563557

[B12] Choi U. Lee C. R. (2019). Distinct roles of outer membrane porins in antibiotic resistance and membrane integrity in *Escherichia coli*. *Front. Microbiol.* 10:953. 10.3389/fmicb.2019.00953 31114568 PMC6503746

[B13] Delcour A. H. (2009). Outer membrane permeability and antibiotic resistance. *Biochim. Biophys. Acta* 1794 808–816. 19100346 10.1016/j.bbapap.2008.11.005 PMC2696358

[B14] Espinosa R. F. Rumi V. Marchisio M. Cejas D. Radice M. Vay C. (2018). Fast and easy detection of CMY-2 in *Escherichia coli* by direct MALDI-TOF mass spectrometry. *J. Microbiol. Methods* 148 22–28. 10.1016/j.mimet.2018.04.001 29621582

[B15] Ferenci T. (2005). Maintaining a healthy SPANC balance through regulatory and mutational adaptation. *Mol. Microbiol.* 57 1–8. 10.1111/j.1365-2958.2005.04649.x 15948944

[B16] Fernandez L. Hancock R. E. (2012). Adaptive and mutational resistance: role of porins and efflux pumps in drug resistance. *Clin. Microbiol. Rev.* 25 661–681. 10.1128/CMR.00043-12 23034325 PMC3485749

[B17] Foudraine D. E. Dekker L. J. M. Strepis N. Bexkens M. L. Klaassen C. H. W. Luider T. M. (2019). Accurate detection of the four most prevalent carbapenemases in *E. coli* and *K. pneumoniae* by high-resolution mass spectrometry. *Front. Microbiol.* 10:2760. 10.3389/fmicb.2019.02760 31849899 PMC6901907

[B18] Foudraine D. E. Strepis N. Klaassen C. H. W. Raaphorst M. N. Verbon A. Luider T. M. (2021a). Rapid and accurate detection of aminoglycoside modifying enzymes and 16S ribosomal RNA methyltransferases by targeted LC-MS/MS. *J. Clin. Microbiol.* 59:e0046421. 10.1128/JCM.00464-21 33910961 PMC8218766

[B19] Foudraine D. E. Strepis N. Stingl C. Ten Kate M. T. Verbon A. Klaassen C. H. W. (2021b). Exploring antimicrobial resistance to beta-lactams, aminoglycosides and fluoroquinolones in *E. coli* and *K. pneumoniae* using proteogenomics. *Sci. Rep.* 11:12472. 10.1038/s41598-021-91905-w 34127720 PMC8203672

[B20] Girlich D. Poirel L. Nordmann P. (2009). CTX-M expression and selection of ertapenem resistance in *Klebsiella pneumoniae* and *Escherichia coli*. *Antimicrob. Agents Chemother.* 53 832–834. 10.1128/AAC.01007-08 19029330 PMC2630641

[B21] Goessens W. H. van der Bij A. K. van Boxtel R. Pitout J. D. van Ulsen P. Melles D. C. (2013). Antibiotic trapping by plasmid-encoded CMY-2 beta-lactamase combined with reduced outer membrane permeability as a mechanism of carbapenem resistance in *Escherichia coli*. *Antimicrob. Agents Chemother.* 57 3941–3949. 10.1128/AAC.02459-12 23733461 PMC3719783

[B22] Grundmann H. Glasner C. Albiger B. Aanensen D. M. Tomlinson C. T. Andrasevic A. T. (2017). Occurrence of carbapenemase-producing *Klebsiella pneumoniae* and *Escherichia coli* in the European survey of carbapenemase-producing *Enterobacteriaceae* (EuSCAPE): a prospective, multinational study. *Lancet Infect. Dis.* 17 153–163. 10.1016/S1473-3099(16)30257-2 27866944

[B23] Hart P. J. Wey E. McHugh T. D. Balakrishnan I. Belgacem O. (2015). A method for the detection of antibiotic resistance markers in clinical strains of *Escherichia coli* using MALDI mass spectrometry. *J. Microbiol. Methods* 111 1–8. 25633625 10.1016/j.mimet.2015.01.020

[B24] Hassing R. J. Goessens W. H. Zeneyedpour L. Sultan S. van Kampen J. J. Verbon A. (2016). Detection of amino acid substitutions in the GyrA protein of fluoroquinolone-resistant typhoidal *Salmonella* isolates using high-resolution mass spectrometry. *Int. J. Antimicrob. Agents* 47 351–356. 10.1016/j.ijantimicag.2016.01.018 27132191

[B25] Hawkey P. M. Livermore D. M. (2012). Carbapenem antibiotics for serious infections. *BMJ* 344:e3236. 10.1136/bmj.e3236 22654063

[B26] Ho P. L. Cheung Y. Y. Wang Y. Lo W. U. Lai E. L. Chow K. H. (2016). Characterization of carbapenem-resistant *Escherichia coli* and *Klebsiella pneumoniae* from a healthcare region in Hong Kong. *Eur. J. Clin. Microbiol. Infect. Dis.* 35 379–385. 26740321 10.1007/s10096-015-2550-3

[B27] Hu Y. Y. Cai J. C. Zhou H. W. Zhang R. Chen G. X. (2015). Rapid detection of porins by matrix-assisted laser desorption/ionization-time of flight mass spectrometry. *Front. Microbiol.* 6:784. 10.3389/fmicb.2015.00784 26300858 PMC4524100

[B28] Idelevich E. A. Becker K. (2019). How to accelerate antimicrobial susceptibility testing. *Clin. Microbiol. Infect.* 25 1347–1355. 10.1016/j.cmi.2019.04.025 31055166

[B29] Intelicato-Young J. Fox A. (2013). Mass spectrometry and tandem mass spectrometry characterization of protein patterns, protein markers and whole proteomes for pathogenic bacteria. *J. Microbiol. Methods* 92 381–386. 10.1016/j.mimet.2013.01.004 23318550

[B30] Iovleva A. Doi Y. (2017). Carbapenem-resistant *Enterobacteriaceae*. *Clin. Lab. Med.* 37 303–315. 28457352 10.1016/j.cll.2017.01.005 PMC5412586

[B31] Jacoby G. A. (2009). AmpC beta-lactamases. *Clin. Microbiol. Rev.* 22 161–182. 19136439 10.1128/CMR.00036-08 PMC2620637

[B32] Jaffe A. Chabbert Y. A. Semonin O. (1982). Role of porin proteins OmpF and OmpC in the permeation of beta-lactams. *Antimicrob. Agents Chemother.* 22 942–948. 10.1128/AAC.22.6.942 6760806 PMC185697

[B33] Knopp M. Andersson D. I. (2015). Amelioration of the fitness costs of antibiotic resistance due to reduced outer membrane permeability by upregulation of alternative Porins. *Mol. Biol. Evol.* 32 3252–3263. 10.1093/molbev/msv195 26358402

[B34] Laemmli U. K. (1970). Cleavage of structural proteins during the assembly of the head of bacteriophage T4. *Nature* 227 680–685. 10.1038/227680a0 5432063

[B35] Livermore D. M. (1995). beta-Lactamases in laboratory and clinical resistance. *Clin. Microbiol. Rev.* 8 557–584. 10.1128/CMR.8.4.557 8665470 PMC172876

[B36] Mammeri H. Guillon H. Eb F. Nordmann P. (2010). Phenotypic and biochemical comparison of the carbapenem-hydrolyzing activities of five plasmid-borne AmpC beta-lactamases. *Antimicrob. Agents Chemother.* 54 4556–4560. 10.1128/AAC.01762-09 20733047 PMC2976168

[B37] Mammeri H. Nordmann P. Berkani A. Eb F. (2008). Contribution of extended-spectrum AmpC (ESAC) beta-lactamases to carbapenem resistance in *Escherichia coli*. *FEMS Microbiol. Lett.* 282 238–240. 10.1111/j.1574-6968.2008.01126.x 18371063

[B38] Miller S. Humphries R. M. (2016). Clinical laboratory detection of carbapenem-resistant and carbapenemase-producing *Enterobacteriaceae*. *Expert Rev. Anti Infect. Ther.* 14 705–717. 10.1080/14787210.2016.1206815 27348447

[B39] Naas T. Oueslati S. Bonnin R. A. Dabos M. L. Zavala A. Dortet L. (2017). Beta-lactamase database (BLDB) – structure and function. *J. Enzyme Inhib. Med. Chem.* 32 917–919. 10.1080/14756366.2017.1344235 28719998 PMC6445328

[B40] Nikaido H. (2003). Molecular basis of bacterial outer membrane permeability revisited. *Microbiol. Mol. Biol. Rev.* 67 593–656. 10.1128/MMBR.67.4.593-656.2003 14665678 PMC309051

[B41] Nordmann P. Poirel L. (2019). Epidemiology and diagnostics of carbapenem resistance in gram-negative bacteria. *Clin. Infect. Dis.* 69 S521–S528. 10.1093/cid/ciz824 31724045 PMC6853758

[B42] Nordmann P. Naas T. Poirel L. (2011). Global spread of carbapenemase-producing *Enterobacteriaceae*. *Emerg. Infect. Dis.* 17 1791–1798. 22000347 10.3201/eid1710.110655 PMC3310682

[B43] Nuramrum S. Chanawong A. Lunha K. Lulitanond A. Sangka A. Wilailuckana C. (2017). Molecular characterization of carbapenemase-nonproducing clinical isolates of *Escherichia coli* (from a Thai University Hospital) with reduced carbapenem susceptibility. *Jpn. J. Infect. Dis.* 70 628–634. 10.7883/yoken.JJID.2017.156 28890516

[B44] Oteo J. Delgado-Iribarren A. Vega D. Bautista V. Rodriguez M. C. Velasco M. (2008). Emergence of imipenem resistance in clinical *Escherichia coli* during therapy. *Int. J. Antimicrob. Agents* 32 534–537. 10.1016/j.ijantimicag.2008.06.012 18775649

[B45] Oviano M. Bou G. (2019). Matrix-assisted laser desorption ionization-time of flight mass spectrometry for the rapid detection of antimicrobial resistance mechanisms and beyond. *Clin. Microbiol. Rev.* 32:e00037–18. 10.1128/CMR.00037-18 30487165 PMC6302359

[B46] Pages J. M. James C. E. Winterhalter M. (2008). The porin and the permeating antibiotic: a selective diffusion barrier in Gram-negative bacteria. *Nat. Rev. Microbiol.* 6 893–903. 10.1038/nrmicro1994 18997824

[B47] Paltansing S. Kraakman M. van Boxtel R. Kors I. Wessels E. Goessens W. (2015). Increased expression levels of chromosomal AmpC beta-lactamase in clinical *Escherichia coli* isolates and their effect on susceptibility to extended-spectrum cephalosporins. *Microb. Drug Resist.* 21 7–16. 10.1089/mdr.2014.0108 25188329

[B48] Pandey N. Cascella M. (2020). *Beta Lactam Antibiotics*. *Book Accesion: NBK545311.* Available online at: https://www.ncbi.nlm.nih.gov/pubmed/31424895 (accessed April 12, 2021). 31424895

[B49] Perez-Perez F. J. Hanson N. D. (2002). Detection of plasmid-mediated AmpC beta-lactamase genes in clinical isolates by using multiplex PCR. *J. Clin. Microbiol.* 40 2153–2162. 10.1128/JCM.40.6.2153-2162.2002 12037080 PMC130804

[B50] Perez-Riverol Y. Csordas A. Bai J. Bernal-Llinares M. Hewapathirana S. Kundu D. J. (2019). The PRIDE database and related tools and resources in 2019: improving support for quantification data. *Nucleic Acids Res.* 47 D442–D450. 10.1093/nar/gky1106 30395289 PMC6323896

[B51] Renault M. Tommassen-van Boxtel R. Bos M. P. Post J. A. Tommassen J. Baldus M. (2012). Cellular solid-state nuclear magnetic resonance spectroscopy. *Proc. Natl. Acad. Sci. U.S.A.* 109 4863–4868. 10.1073/pnas.1116478109 22331896 PMC3323964

[B52] Tamma P. D. Simner P. J. (2018). Phenotypic detection of carbapenemase-producing organisms from clinical isolates. *J. Clin. Microbiol.* 56:e01140–18. 10.1128/JCM.01140-18 30158194 PMC6204673

[B53] Tangden T. Adler M. Cars O. Sandegren L. Lowdin E. (2013). Frequent emergence of porin-deficient subpopulations with reduced carbapenem susceptibility in ESBL-producing *Escherichia coli* during exposure to ertapenem in an in vitro pharmacokinetic model. *J. Antimicrob. Chemother.* 68 1319–1326. 10.1093/jac/dkt044 23478794

[B54] Tian X. Zheng X. Sun Y. Fang R. Zhang S. Zhang X. (2020). Molecular mechanisms and epidemiology of carbapenem-resistant *Escherichia coli* isolated from chinese patients during 2002-2017. *Infect. Drug Resist.* 13 501–512. 32110061 10.2147/IDR.S232010 PMC7035005

[B55] Traczewski M. M. Carretto E. Canton R. Moore N. M. Carba R. S. T. (2018). Multicenter evaluation of the xpert carba-r assay for detection of carbapenemase genes in gram-negative isolates. *J. Clin. Microbiol.* 56:e00272–18. 10.1128/JCM.00272-18 29848561 PMC6062815

[B56] van Boxtel R. Wattel A. A. Arenas J. Goessens W. H. Tommassen J. (2017). Acquisition of carbapenem resistance by plasmid-encoded-AmpC-expressing *Escherichia coli*. *Antimicrob. Agents Chemother.* 61 e1413–e1416. 10.1128/AAC.01413-16 27799202 PMC5192137

[B57] Vergalli J. Bodrenko V. I. Masi M. Moynie L. Acosta-Gutierrez S. Naismith J. H. (2020). Porins and small-molecule translocation across the outer membrane of Gram-negative bacteria. *Nat. Rev. Microbiol.* 18 164–176. 10.1038/s41579-019-0294-2 31792365

[B58] Wan Nur Ismah W. A. K. Takebayashi Y. Findlay J. Heesom K. J. Jimenez-Castellanos J. C. Zhang J. (2018). Prediction of fluoroquinolone susceptibility directly from whole-genome sequence data by using liquid chromatography-tandem mass spectrometry to identify mutant genotypes. *Antimicrob. Agents Chemother.* 62:e01814–17. 10.1128/AAC.01814-17 29263066 PMC5826109

[B59] Wang H. Chen Y. Strich J. R. Drake S. K. Youn J. H. Rosenberg A. Z. (2019). Rapid detection of colistin resistance protein MCR-1 by LC-MS/MS. *Clin. Proteomics* 16:8. 10.1186/s12014-019-9228-2 30890899 PMC6390366

[B60] Wang H. Drake S. K. Youn J. H. Rosenberg A. Z. Chen Y. Gucek M. (2017). Peptide markers for rapid detection of KPC carbapenemase by LC-MS/MS. *Sci. Rep.* 7:2531. 10.1038/s41598-017-02749-2 28566732 PMC5451396

[B61] Wick R. R. Judd L. M. Gorrie C. L. Holt K. E. (2017). Unicycler: resolving bacterial genome assemblies from short and long sequencing reads. *PLoS Comput. Biol.* 13:e1005595. 10.1371/journal.pcbi.1005595 28594827 PMC5481147

[B62] Wozniak A. Villagra N. A. Undabarrena A. Gallardo N. Keller N. Moraga M. (2012). Porin alterations present in non-carbapenemase-producing *Enterobacteriaceae* with high and intermediate levels of carbapenem resistance in Chile. *J. Med. Microbiol.* 61 1270–1279. 10.1099/jmm.0.045799-0 22700549

[B63] Yang Q. Wang H. Sun H. Chen H. Xu Y. Chen M. (2010). Phenotypic and genotypic characterization of *Enterobacteriaceae* with decreased susceptibility to carbapenems: results from large hospital-based surveillance studies in China. *Antimicrob. Agents Chemother.* 54 573–577. 10.1128/AAC.01099-09 19805565 PMC2798477

