# Supplementary material for: Liquid Chromatography-Tandem Mass Spectrometry Analysis Demonstrates a Decrease in Porins and Increase in CMY-2 β-Lactamases in Escherichia coli Exposed to Increasing Concentrations of Meropenem
Source: Front Microbiol. 2022 Feb 28;13:793738. doi: 10.3389/fmicb.2022.793738 (PMC8918824; doi:10.3389/fmicb.2022.793738)
Supplement: Supplementary file 1 [file Table_1.DOCX]

**Table S1**: All candidate peptides that were selected for the detection of the beta-lactamases cAmpC and CMY-2-like, and the porins OmpC and OmpF in *E. coli*.

| **Protein** | **Peptide** | **Coverage of sequences from NCBI blast** | **Coverage of annotated sequences** | **Mass (m/z)** |
| --- | --- | --- | --- | --- |
| cAmpC | YWPELTAK | 1124 / 1179 | 392 / 405 | 504,26347 |
|  | KQPVTQQTLFELGSVSK | 1170 / 1179 | 367 / 405 | 945,52019 |
|  | QPVTQQTLFELGSVSK | 1170 / 1179 | 367 / 405 | 881,47271 |
|  | SSSDLLR | 1167 / 1179 | 371 / 405 | 389,20869 |
|  | TGATGGFGSYVAFIPEK | 1168 / 1175 | 365 / 405 | 851,42777 |
|  | NYPNPAR | 1170 / 1173 | 363 / 405 | 416,20903 |
|  | PYYFTWGYADIAKK | 1147 / 1179 | 352 / 405 | 797,88247 |
|  | PYYFTWGYADIAK | 1147 / 1179 | 352 / 405 | 861,92995 |
| CMY-2-like | PYYFTWGK | 938 / 950 | 152 / 154 | 531,25819 |
|  | TFNGVLGGDAIAR | 925 / 950 | 150 / 154 | 645,84368 |
|  | TGSTGGFGSYVAFVPEK | 893 / 925 | 149 / 154 | 852,41740 |
|  | TEQQIADIVNR | 900 / 924 | 148 / 154 | 643,83859 |
|  | LSDPVTK | 877 / 950 | 139 / 154 | 380,21599 |
|  | ADSIINGSDSK | 868 / 950 | 135 / 154 | 553,76984 |
| OmpC | VAFAGLK | 1202 / 1202 |  | 353,21833 |
|  | AETYTGGLK | 1201 / 1202 |  | 470,24273 |
|  | VGSLGWANK | 1170 / 1202 |  | 466,25343 |
|  | YDANNIYLAAQYTQTYNATR | 1167 / 1202 |  | 1177,55602 |
|  | FQDVGSFDYGR | 1148 / 1202 |  | 645,79111 |
|  | GNGFATYR | 1117 / 1202 |  | 443,21431 |
| OmpF | PSIAYTK | 1126 / 1127 |  | 390,21853 |
|  | AVGLHYFSK | 1122 / 1125 |  | 511,27691 |
|  | VGGVATYR | 1127 / 1127 |  | 411,72705 |
|  | KAEQWATGLK | 1126 / 1127 |  | 566,31148 |
|  | AEQWATGLK | 1126 / 1127 |  | 502,26400 |
|  | YDANNIYLAANYGETR | 1124 / 1127 |  | 924,43157 |
|  | TQDVLLVAQYQFDFGLR | 1119 / 1127 |  | 1007,02564 |

All peptides were measured with a charge of 2+. Coverage of each peptide was assessed by comparing variant sequences of each resistance mechanism obtained by BLASTn searches using the reference sequences and the NCBI nucleotide collection (nr/nt) database. In addition, coverage of the annotated sequences from the Beta-Lactamase Database (Naas et al., 2017) was also assessed.
