# Supplementary figures and images for: Liquid Chromatography-Tandem Mass Spectrometry Analysis Demonstrates a Decrease in Porins and Increase in CMY-2 β-Lactamases in Escherichia coli Exposed to Increasing Concentrations of Meropenem
Source: Front Microbiol. 2022 Feb 28;13:793738. doi: 10.3389/fmicb.2022.793738 (PMC8918824; doi:10.3389/fmicb.2022.793738)

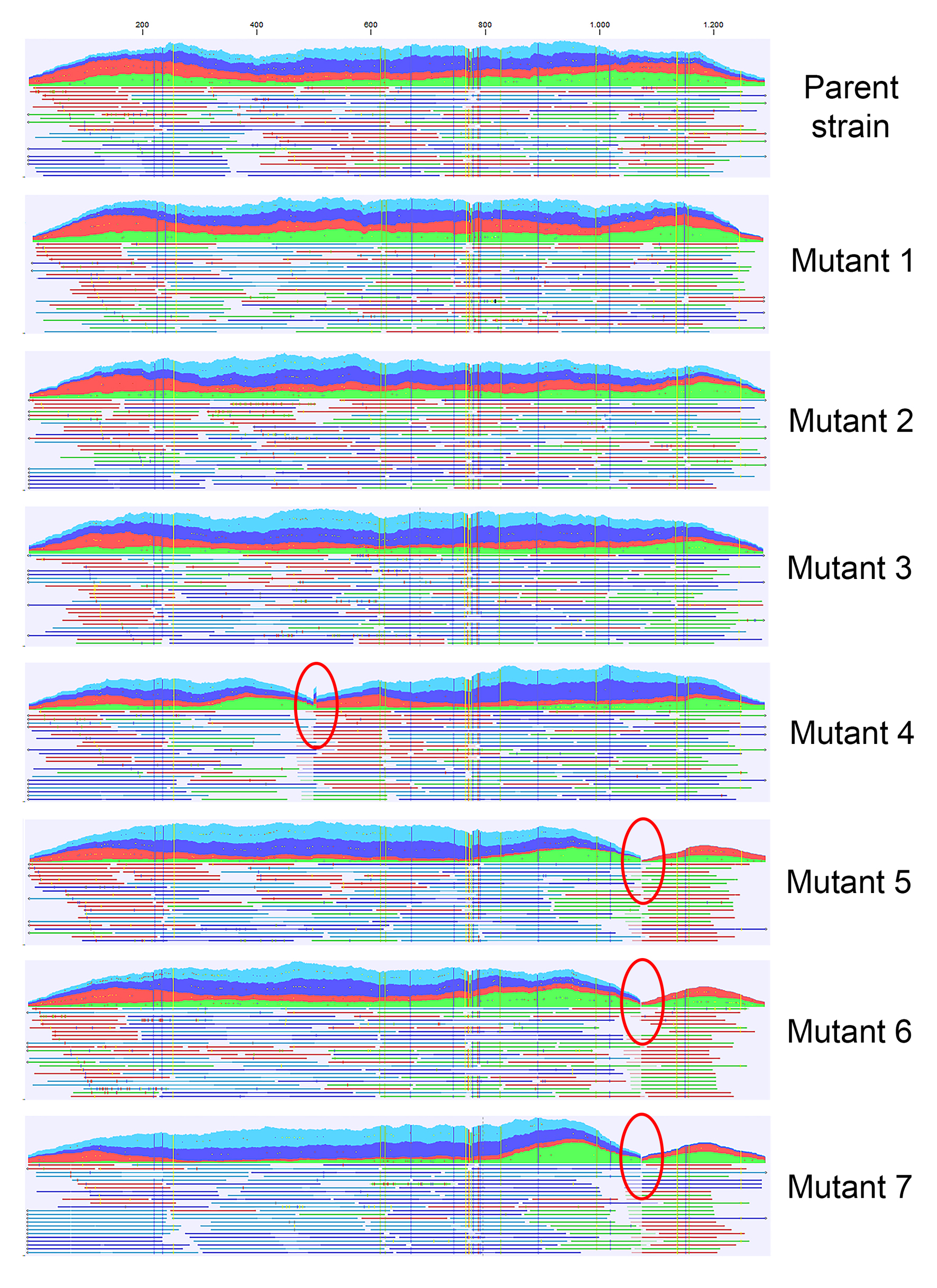

Supplement: Supplementary Figure 1 — Application of read-mapping to check the structural integrity of genes. The example shows the read-mapping results of the B2591 parent strain and its mutants for the ompF gene using the gene from E. coli MG1655 plus 100 nucleotide flanking sequences as a reference. Colors in the cumulative coverage graph and from individual reads are as follows. Dark-blue: forward paired read; light blue: reverse paired read; green: forward unpaired read; red: reverse unpaired read. The vertical colored lines indicate sequence variations relative to the reference sequence. Below the cumulative coverage graph, a fraction of the individual reads are shown. The red oval indicates regions where the structural integrity of the gene was affected. In mutant 4, the gene was affected at a different position than in mutants 5–7. [file Image_1.JPEG]
